# Optimization of sperm RNA processing for developmental research

**DOI:** 10.1038/s41598-020-68486-1

**Published:** 2020-07-14

**Authors:** Won-Ki Pang, Saehan Kang, Do-Yeal Ryu, Md Saidur Rahman, Yoo-Jin Park, Myung-Geol Pang

**Affiliations:** Department of Animal Science and Technology and BET Research Institute, Chung-Ang University, Anseong, Gyeonggi-do 17546 Republic of Korea

**Keywords:** Cell biology, Developmental biology, Biomarkers

## Abstract

Recent studies have demonstrated the significance of sperm RNA function as a transporter of important information directing the course of life. To determine the message contained in sperm RNA, it is necessary to optimize transcriptomic research tools. The current study was performed to optimize the processing of sperm RNA from sample storage to quantitative real-time PCR and assess the corresponding method with to evaluate male fertility and its representative markers, equatorin (EQTN) and peroxiredoxin (PRDX). Following successive steps of the Minimum Information for Publication of Quantitative Real-Time PCR Experiments guidelines, several options were compared using boar spermatozoa. To evaluate the optimized procedures, the relationship between mRNA expression of EQTN and PRDX in spermatozoa and the fertility (litter size) of 20 individual boars was assessed. Unexpectedly, DNase treatment during RNA isolation had the deleterious effect by decreasing the RNA concentration by 56% and eliminating the correlation between EQTN and PRDX4 mRNA expression and male fertility. Moreover, when sperm RNA was processed using the corresponding method, the results showed the highest exon sequence expression, male fertility prediction power, and consistency. This optimized protocol for predicting male fertility can be used to study the transport of messages directing the life course from spermatozoon to offspring.

## Introduction

A spermatozoon is a haploid gamete that conveys paternal genetic information to offspring. During the differentiation to spermatozoon, the transcriptional activity of the cell shuts down^1^. Although the condensed nucleus of spermatozoa is thought to be in a transcriptionally inert state, sperm RNA was identified in many mammalian species, such as human^2^, bovine^3^, porcine^4^, equine^5^, and rodent^6^. Several plausible hypothesis and experimentation/observations on sperm RNA function, *i.e.* contribution during fertilization by delivering novel paternal RNA to the oocyte^7–9^ and signaling in mitochondrial translation^10^, are widely known. Moreover, the possibility of using sperm RNA as a marker for diagnosis of male fertility and infertility has recently gained attention^11–13^. Recent studies suggested that many phenotypes from parent generation are transferred to the progeny through sperm RNA^14,15^. Additionally, sperm RNAs were suggested to relay the message to one’s life course, providing the opportunity to study health at key stages of life from preconception to offspring^16^. However, despite abundant evidences on the functional significance of sperm RNAs, the contribution on sperm biology remains controversial^17,18^ and efforts are needed to optimize transcriptomic research tools. This may lead to improvements in the understanding of the underlying mechanisms of how spermatozoon affects the course of life.

The main transcriptomic research tools currently being used are RNA sequencing (RNA-seq) and reverse transcription quantitative real-time polymerase chain reaction (RT-qPCR)^19,20^. To facilitate sperm RNA research, these protocols require some modifications because of the complexity of sperm biology, including the lower RNA levels compared to other cell types^21^ and disulfide bond-rich protamine-packed chromatin structure that resists lysis during RNA isolation^22^. Moreover, most RNAs except for a portion of the nucleus exists in a highly fragmented state in spermatozoa^23^. Thus, isolation of intact sperm RNA is important and has been widely studied^6,22,24^. As a result, various transcriptomic studies of spermatozoa related to sperm function, male fertility, idiopathic infertility, sperm-oocyte fusion, and inheritance of the paternal phenotype have been conducted^16,25,26^.

Despite the advantages of RNA-seq, such as its high resolution on total aspects of biological sample, it has several limitations including low specificity, sensitivity, and reproducibility when used for the prognosis and diagnosis of specific phenotypes^19^. Compare to RNA-seq, RT-qPCR is useful for screening specific target genes for predicting prognosis and diagnosing disease, phenotypes, and other biological aspects in various samples^27^. According to Minimum Information for Publication of Quantitative Real-Time PCR Experiments (MIQE) guidelines, the use of RNA stabilizing solution and DNase is essential^28^. Many researchers perform DNase treatment to increase the RNA ratio in the sample. Additionally, an RNA-stabilizing solution (*e.g.* RNAlater) is widely used to stabilize and protect cellular RNA because of its immediate RNase inactivation characteristics^29^. However, it is not examined that whether these treatments could be used to improve RT-qPCR results in spermatozoa or not.

Compare to proteomic markers in sperm^30,31^, only a few studies have reported that sperm mRNA expression is correlated with male fertility^32–34^. One of the reasons is the protocols for accurate prognosis and diagnosis of male fertility have not been optimized. Therefore, we selected several steps that may affect sperm RNA and RT-qPCR data according to MIQE guidelines^28^. Sperm sample storage, RNA isolation, cDNA synthesis, and RT-qPCR conditions were compared to maximize the power of predicting male fertility using mRNA markers. We used a porcine model to test these conditions because of its large available amount of fertility data from artificial insemination (AI)^35^ and well-established genetic traits related to fertility, particularly in large white pigs^36^. The correlation between mRNA expression in spermatozoa and the phenotype related with the life course from spermatozoon through offspring was evaluated using RNA markers related to litter size, equatorin (EQTN)^37,38^ and peroxiredoxin (PRDX)^39,40^ after improving sperm RNA preparation and RT-qPCR. The EQTN and PRDX4 are related to sperm acrosome function^38,40^ and ample evidence suggests the levels of EQTN and PRDX4 protein in spermatozoa are correlated with male fertility^37,39^. Therefore, in this study, we try to establish whether transcript levels of an EQTN/PRDX4 can be used as proxies for the corresponding protein levels.

## Results

### Effect of sperm processing condition on isolated sperm RNA

**Figure 1:**
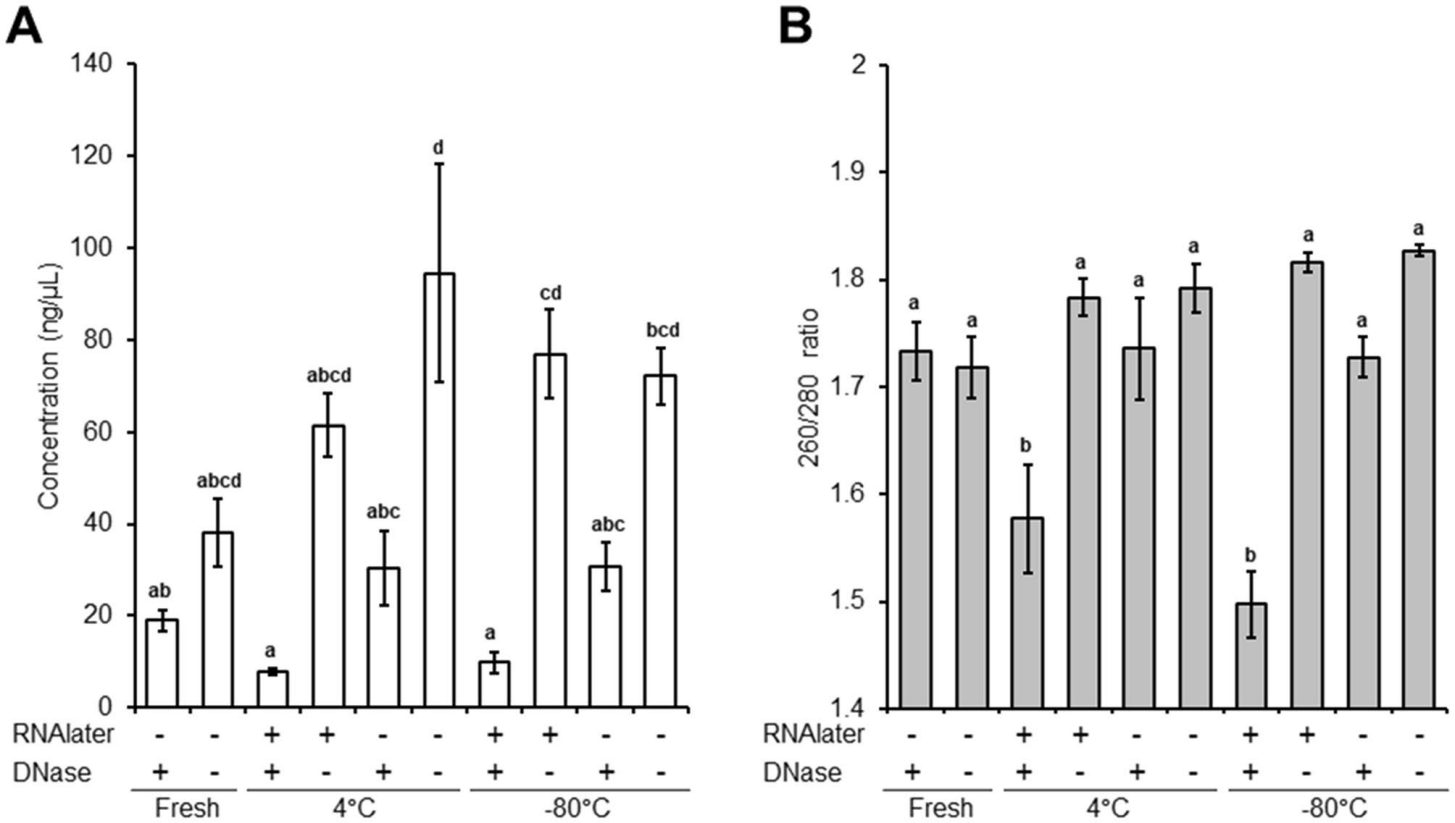
Effect of storage conditions and DNase treatment on isolated RNA quantity and quality. (**A**) RNA quantity is shown as the concentration (ng/µL) of isolated RNA in each treatment. (**B**) RNA quality is shown as 260/280 ratio of RNA. a–d *P* < 0.05 tested by one-way analysis of variance. Post-hoc test was Duncan in RNA quantity and Tukey’s method in RNA quality. All data are expressed as the mean ± SE.

To test the effect of storage conditions on isolated sperm RNA, washed spermatozoa were snap-frozen in liquid nitrogen (LN_2_) or treated with RNAlater and stored in 4 °C or− 80 °C for 7 days. Subsequently, sperm RNAs were isolated with DNase treated or non-treated protocol. Neither RNAlater nor DNase treatment alone affected RNA quantity (Fig. 1A) and quality (260/280 ratio, Fig. 1B). However, when RNA was isolated with DNase from RNAlater-treated samples, the 260/280 ratio was significantly decreased compared to the other groups (Fig. 1B) and its concentration was lower compared to isolated RNA without DNase (Fig. 1A). Furthermore, the average RNA concentration of 20 individual sperm samples was 56% lower when treated with DNase (44.28 ± 8.12 ng/µL) during isolation compared to samples not treated with DNase (101.7 ± 13.5 ng/µL, Table 1). Despite the deleterious effect of DNase treatment on the concentration of RNA, the average quality was same in both RNA isolated with (1.79 ± 0.03) or without DNase (1.80 ± 0.01, Table 1).

**Table 1:** Isolated RNA from randomly selected 20 boar spermatozoa.

Sample name	Litter size	Isolated RNA without DNase		Isolated RNA with DNase	
		Concentration (ng/µL)	260/280 ratio	Concentration (ng/µL)	260/280 ratio
1	14.15 ± 0.79	41.25	1.74	67.72	1.86
2	13.44 ± 0.68	153.97	1.86	35.80	1.79
3	13.30 ± 0.95	75.89	1.74	74.12	1.93
4	13.15 ± 0.79	140.45	1.87	123.00	1.85
5	13.14 ± 0.55	146.45	1.87	58.04	1.86
6	13.10 ± 0.99	88.29	1.83	17.00	1.54
7	12.90 ± 1.06	152.45	1.85	28.12	1.81
8	12.82 ± 0.80	25.81	1.70	129.56	1.93
9	12.79 ± 0.94	109.57	1.79	96.12	1.91
10	12.75 ± 1.28	197.57	1.82	21.40	1.89
11	12.67 ± 0.72	255.65	1.83	13.40	1.61
12	12.65 ± 0.64	29.33	1.77	21.08	1.84
13	12.54 ± 0.75	58.93	1.83	42.84	1.84
14	12.40 ± 1.72	93.89	1.82	33.64	1.88
15	12.38 ± 0.85	33.89	1.58	10.12	1.80
16	12.25 ± 0.77	83.89	1.82	33.96	1.84
17	12.08 ± 0.51	108.29	1.80	37.32	1.77
18	11.81 ± 0.71	54.45	1.80	18.28	1.55
19	11.75 ± 1.16	128.37	1.85	13.64	1.55
20	11.35 ± 0.92	56.37	1.78	10.36	1.72
Average	12.67 ± 0.14	101.7 ± 13.5*	1.80 ± 0.01	44.28 ± 8.12	1.79 ± 0.03

The male fertility data (total litter size) and RNA quantity and quality of isolated RNA with or without DNase in 20 randomly selected boar spermatozoa. *P < 0.05, calculated using two-tailed Student's *t*-test.

### Whole sperm RNA processing method on RT-qPCR outcome

**Figure 2:**
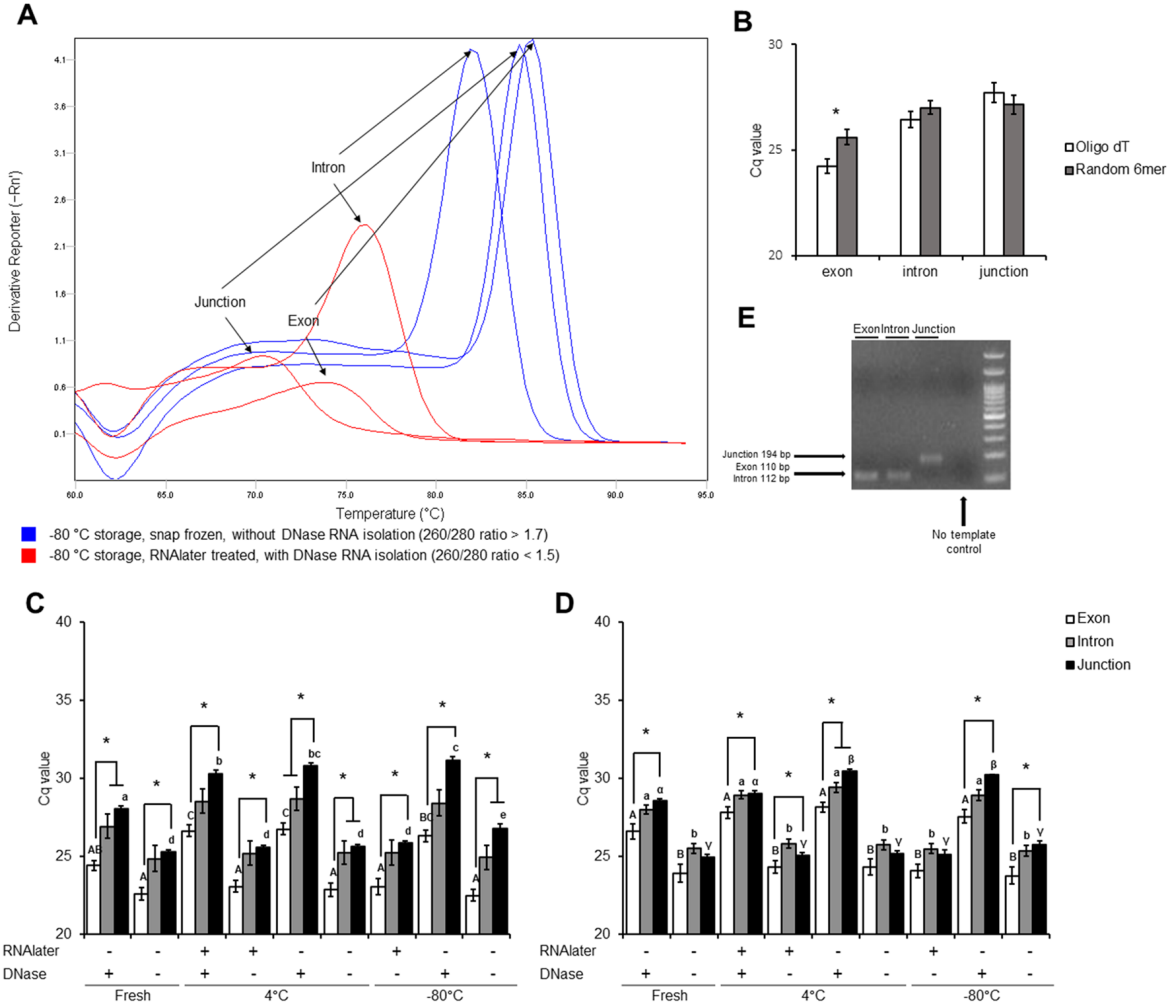
Effect of total sperm RNA processing method on RT-qPCR. (**A**) Melting curve of− 80 °C storage, snap-frozen, without DNase RNA isolation as a representative of normal RT-qPCR data from good quality RNA (260/280 ratio > 1.7) and− 80 °C storage, RNAlater-treated, with DNase RNA isolation as a representative of unnormal RT-qPCR data from bad quality RNA (260/280 ratio < 1.5). (**B**) Average Cq value of GAPDH exon, intron, and junction primers in synthesized cDNA with oligo dT and random hexamer primers. *P < 0.05 tested by Student’s *t*-test (**C**) Cq value of GAPDH exon, intron, and junction primers in synthesized cDNA with oligo dT in each method. *P < 0.05 tested by one-way analysis of variance among Cq value of each primers. A-C, a-d *P* < 0.05 tested by one-way analysis of variance among treatments (**D**) Cq value of GAPDH exon, intron, and junction primers in synthesized cDNA with random hexamer in each method. *P < 0.05 tested by one-way analysis of variance among Cq value of each primers. A–B, a–b, α–γ *P* < 0.05 tested by one-way analysis of variance among treatments (**E**) Representative gel electrophoresis image of RT-qPCR product in− 80 °C storage, snap-frozen, without DNase RNA isolation method with no template control. This image was cropped from Supplementary Figure S1A online. (**B**–**D**) All data are expressed as the mean ± SE.

To compare each sperm RNA processing method, cDNA was synthesized by using oligo dT or random hexamer primers and RT-qPCR was performed with exon, intron, and junction primers of the glyceraldehyde 3-phosphate dehydrogenase (GAPDH) gene. The cDNA synthesis efficiency for oligo dT and random hexamer showed no significant difference (Supplementary Fig. S1 online). No amplification was observed in both oligo dT and random hexamers no template cDNA (Supplementary Fig. S2 online). The average Cq value difference of EQTN, PRDX4, and GAPDH between no reverse transcriptase and positive control was 7.0 ± 0.4, 11.0 ± 0.3, and 10.6 ± 0.3, respectively (Supplementary Fig. S3A online). The average Cq value difference of EQTN, PRDX4, and GAPDH between no reverse transcriptase and cDNA from sperm RNA was 4.5 ± 0.4, 4.2 ± 0.3, and 6.4 ± 1.3, respectively (Supplementary Fig. S3B online). The RT-qPCR data of isolated RNA with DNase from − 80 °C stored after RNAlater treatment was excluded from further statistical analysis because its melting temperature differed in melting curve analysis and showed a decreased amplification peak compared to the other samples (Fig. 2A). The average Cq value of exon primers between oligo dT and random hexamers showed a significant difference (Fig. 2B). The GAPDH RT-qPCR data showed that the three methods (1. isolated RNA from fresh spermatozoa with DNase. 2. isolated RNA from 4 °C stored spermatozoa without RNAlater and DNase treatment. 3. isolated RNA from− 80 °C stored spermatozoa without RNAlater and DNase treatment.) in oligo dT cDNA and only one method (isolated RNA from 4 °C stored spermatozoa without RNAlater and with DNase treatment) in random hexamer cDNA showed a lower Cq value in the exon primer compared to using both intron and junction primers (Fig. 2C,D). The sizes of the PCR products were 110, 112, and 194 base pairs in the exon, intron, and junction, respectively (Fig. 2E and Supplementary Fig. S4 online).

### Correlation between RT-qPCR data and male fertility

**Figure 3:**
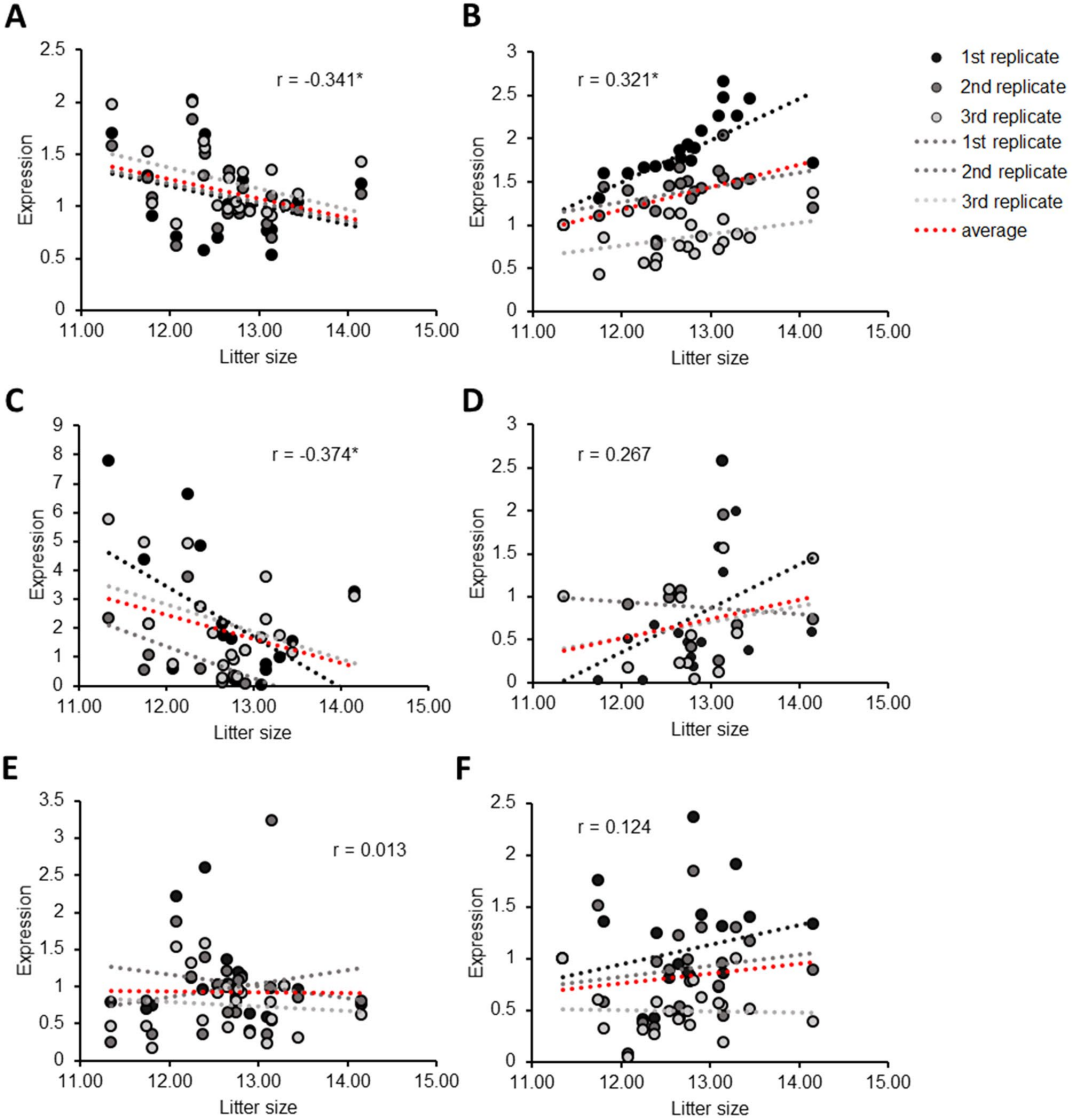
Correlation between EQTN and PRDX4 expression with male fertility. (**A**) linear regression test of EQTN mRNA expression and litter size in isolated RNA without DNase in 20 randomly selected boar spermatozoa. (**B**) Linear regression test of PRDX4 mRNA expression and litter size in isolated RNA without DNase in randomly selected 20 boar spermatozoa. (**C**) Linear regression test of EQTN mRNA expression when gDNA considered with intron primer expression and litter size in isolated RNA without DNase in 20 randomly selected boar spermatozoa. (**D**) Linear regression test of PRDX4 mRNA expression when gDNA was considered with intron primer expression and litter size in isolated RNA without DNase in 20 randomly selected boar spermatozoa. (**E**) Linear regression test of EQTN mRNA expression and litter size in isolated RNA with DNase in 20 randomly selected boar spermatozoa. (**F**) Linear regression test of PRDX4 mRNA expression and litter size in isolated RNA with DNase in 20 randomly selected boar spermatozoa. r, Pearson correlation coefficient; *P < 0.05, calculated using linear regression test.

To evaluate the correlation of RT-qPCR data for each genes and male fertility, the relative expression of genes in 20 individual boar spermatozoa were examined using optimized procedures. According to cDNA synthesis and RT-qPCR data for the GAPDH primer, the oligo dT primer in cDNA synthesis and exon primer of the gene in RT-qPCR were adequate for measuring the expression of mRNA. To rule out the effects of genomic DNA (gDNA), intron primers for each marker were also checked with RT-qPCR. Total cDNAs of 20 individual boar spermatozoa were prepared and the mRNA expression of the EQTN and PRDX4 genes was evaluated. EQTN and PRDX4 expression levels were significantly correlated with litter size when RNA was isolated without DNase and when the Cq value from exon primer RT-qPCR was considered as mRNA expression (rEQTN = − 0.341 and rPRDX4 = 0.321; Fig. 3A,B). When the Cq value of the intron primer was considered to rule out the gDNA factor, only EQTN was significantly correlated with litter size (rEQTN = − 0.374 and rPRDX = 0.267; Fig. 3C,D). In contrast to the data obtained from isolated RNAs without DNase, the relative expression of both EQTN and PRDX4 in DNase-treated RNA showed no correlation with litter size (rEQTN = 0.013 and rPRDX = 0.124; Fig. 3E,F). Additionally, the Cq value from the intron primer of DNase-treated RNA could not be considered to rule out the gDNA factor because there was no difference from the Cq value of the exon primer.

### Assessment of male fertility predicting power of mRNA markers

**Figure 4:**
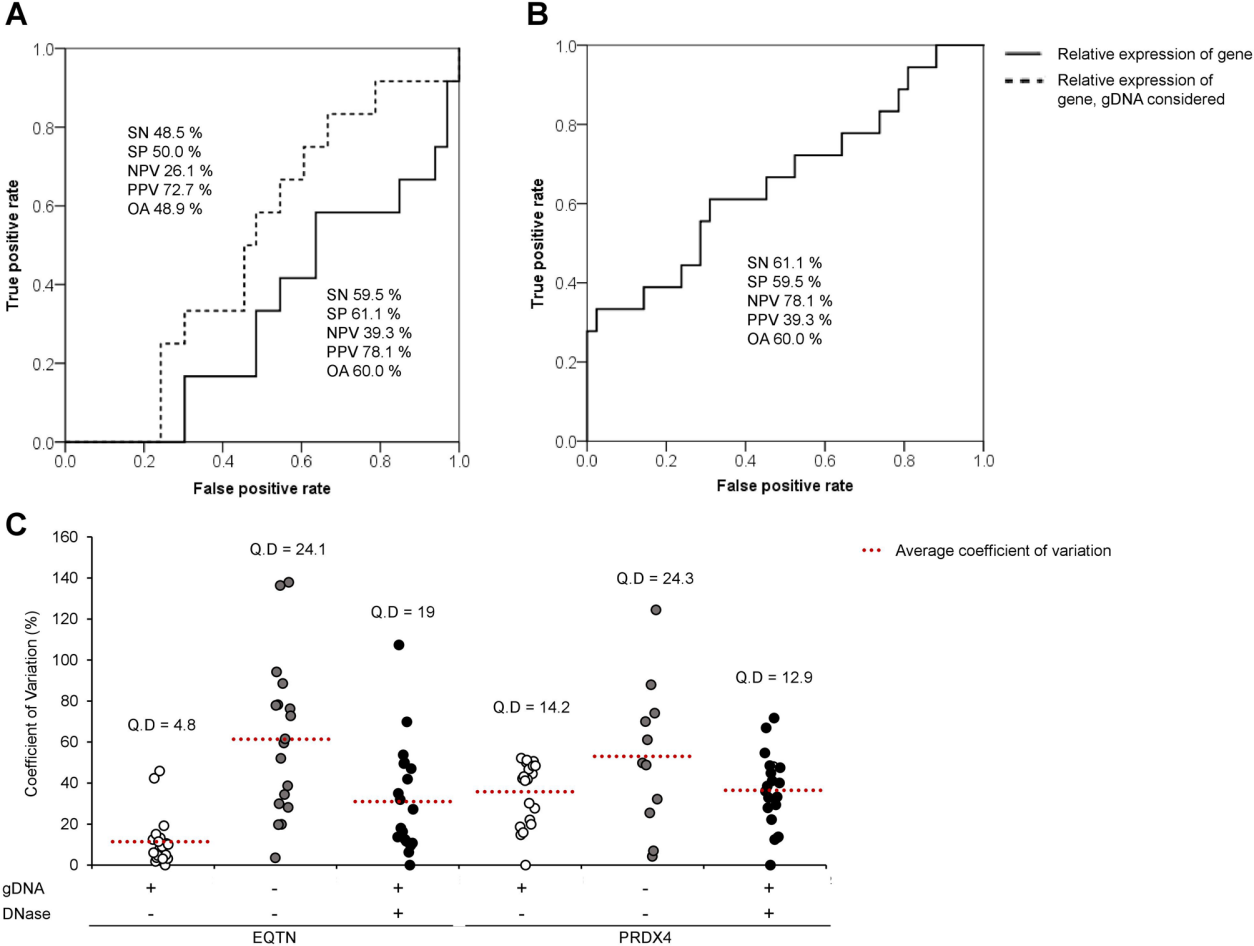
ROC curve of EQTN and PRDX4 expression comparing litter size. (**A**) ROC curve for EQTN mRNA expression when gDNA was considered or not and litter size in isolated RNA without DNase in 20 randomly selected boar spermatozoa. (**B**) ROC curve for PRDX4 mRNA expression and litter size in isolated RNA without DNase in 20 randomly selected boar spermatozoa. (**C**) The coefficient of variation (%) and quartile deviation (Q.D) of EQTN and PRDX4 mRNA expression in each processing method. The cut-off values of relative expression in all treatments were determined based on fixed litter size (13) for comparison among each treatment. Sensitivity (SN) is the percentage of boars showing true-positive results when tested with mRNA expression. Specificity (SP) is the percentage of boars showing true-negative results. The positive predictive value (PPV) is the percentage of boars that tested as positive and simultaneously has a true-positive litter size. The negative predictive value (NPV) is the percentage of boars that tested as negative or simultaneously had a true-negative litter size. OA, Overall accuracy.

To assess the male fertility predicting power of mRNA markers when processed with optimized protocols, ROC curve analysis was performed. The overall accuracies of EQTN and PRDX4 in non-DNase-treated samples were the same (60%; Fig. 4A,B). When the gDNA factor was considered in EQTN relative expression, all predictive values were decreased (sensitivity, specificity, negative predictive value, positive predictive value, and overall accuracy were 59.52%, 61.11%, 39.29%, 78.13%, and 60.00% before gDNA consideration and 48.48%, 50.00%, 26.09%, 72.73%, and 48.89% after gDNA consideration, respectively; Fig. 4A). Interestingly, although the cDNA templates were from same samples, the coefficient of variation and quartile deviation showed different patterns according to DNase treatment and gDNA factor consideration (Fig. 4C).

## Discussion

Despite the critical contribution of sperm RNA to male fertility^26,41^, embryo development^42^, epigenetic inheritance for acquired traits included in the paternal genome^43^, and health^44^, the lack of optimization of transcriptomic research tools prevents a thorough understanding of sperm biology. Therefore, we optimized the processing of sperm RNA from sample storage to RT-qPCR and evaluated the male fertility predicting power of the optimized protocol.

The significance of the porcine model on biomedical research studying a spectrum of human diseases, including obesity, arthritis, cardiovascular disease, and skin and eye conditions are already well known^45,46^. In addition, Several studies emphasize the particular importance of the porcine model in xenotransplantation^47^. In the same vein, the porcine model can also be useful in the study of human male infertility. What makes this possible is the similarity between porcine genome and the human genome and well-organized breeding data from AI^35,48^. Furthermore, many researh groups are conducting comprehensive trascriptomic analysis in boar spermatozoa^49–51^. In the present study, a porcine model was used to evaluate the fertility prediction of sperm RNA.

First, we compared RNAlater treatment to the snap-freezing method in LN_2_ for sample storage step. Many recent studies reported that the effect of RNAlater on isolated RNA depends on the cell type, storage period, and research application^52–55^. To the best of our knowledge, this is the first study to compare the use of RNAlater and snap-freezing for spermatozoa. RNAlater treatment was time-consuming and costly, without beneficially affecting sperm RNA. Thus, treatment with RNAlater was not used in further steps.

For RNA isolation of spermatozoa, a two-step lysis was used. Spermatozoa chromatin is consists of the enriched disulfide bonds, which can only be broken down by detergents (Sodium dodecyl dulfate or sodium lauryl sulfate) and reducing agents (e.g., dithiothreitol or β-mercaptoethanol). To successfully isolate the large number of sperm RNAs in sperm nuclei^21,22^, the sperm head should be fully lysed. Trizol, which contains neither detergents nor reducing agents, cannot lysis sperm head. For the separation of sperm RNA from a cell lysate, chloroform was used. Chloroform is a common and key reagent used for isolation of sperm RNA, both by Trizol-modified methods but also for commercially available RNA extraction kits during phase separation^5,56^.

Generally, DNase is used to rule out gDNA contamination in isolated RNA. However, DNase has harmful effect not only in gDNA but also in concentrations of RNA and small RNAs^57,58^. In this study, DNase affected RNA concentration and quality in RNAlater-treated samples. Although the DNase alone had no significant effect on RNA quality during the optimization procedure, the exon Cq value of DNase-treated samples was similar with the intron value. Based on these studies, we suggested that DNase may induce the degradation of small RNA in spermatozoa during RNA isolation. This could lead to biased interpretation of sperm transcriptomic study. Therefore, later researchers need to reconsider the use of DNase during sperm RNA study.

For the cDNA synthesis step, the effects of oligo dT and random hexamer primers were compared. The size of the PCR product with the junction primer for GAPDH was 194 base pairs, suggesting that most amplicons were from gDNA. Because of this, the Cq value of the exon primer should be significantly lower than that of the intron primer and junction primer for detecting mRNA. In case of fresh sperm sample, DNase treated RNA showed this corresponding expression pattern. The oligo dT primer cDNA, from 4 to − 80 °C snap-frozen storage and without DNase-treated isolated sperm RNA samples also showed this corresponding expression pattern. Thus, snap-frozen samples in LN_2_, with or without DNase RNA isolation and Oligo dT cDNA synthesis, were selected as the optimized sperm processing conditions for predicting male fertility.

To assess the male fertility predicting power of mRNA markers using the optimized protocols, a correlation test was conducted to evaluate the mRNA expression of EQTN and PRDX4 could represent the male fertility data or not. EQTN and PRDX4 exon expression levels were negatively and positively correlated with litter size in isolated RNA without DNase, respectively. In contrast, no correlation between the exon expression of EQTN and PRDX4 from isolated RNA with DNA was observed. As previously noticed, in the isolated RNA with DNase treatment, the expression of the exon sequence was similar or lower than the expression of the intron sequence. In the other word, the portion of RNA in the sample was too low to indicate the correlation between gene expression and male fertility. Laurell et al*.* described a method for ruling out the gDNA factor in cDNA samples using an optimized gDNA-specific ValidPrime assay and gDNA reference sample^59^. Similarly, we directly evaluated the expression of gene intron sequences and attempted to exclude gDNA contamination. Only EQTN was correlated with male fertility when the gDNA factor was considered. To clarify the effect of gDNA factor consideration in male fertility prediction value the cut-off value of EQTN relative expression was settled at fixed litter size (13). The decreased male fertility prediction value in EQTN expression was a major limitation when considering the gDNA factor. Moreover, the losing correlation on PRDX4 and decrease in predictive value of EQTN was corresponded with coefficient of variation and quartile deviation.

In summary, we optimized the processing of sperm RNA for screening male fertility from sample storage to RT-qPCR. The optimized condition involved snap-freezing in LN_2_, without DNase RNA isolation, oligo dT cDNA synthesis, and RT-qPCR with exon primers. The highest exon sequence expression, male fertility prediction power, and consistency were obtained using this method. The mRNA level obtained using the optimized method showed better male fertility predicting power and consistency of data. This study will shed light on medical and industrial use of sperm RNA to evaluate infertility, animal health, and offspring phenotypes. Moreover, the optimized protocol can be used to elucidate the underlying mechanisms of life course research from spermatozoa to the next generation.

## Materials and methods

All procedures involving animals were approved by the Institutional Animal Care and Use Committee of Chung-Ang University (Approval No. 2017-00018) and performed according to the corresponding guidelines. All methods were performed in accordance with the relevant guidelines and regulations.

### Sperm sample preparation

Yorkshire boar semen was provided by Sunjin Co. grand-grand parents farm (Danyang, Korea). The sexually mature (11–23 months) and body weight over 90 kg boars were used to collect semen sample. For the laboratory work, the semen samples were collected once in summer season (June–July). The ejaculates of boar were obtained by gloved-hand technique^60^ and collected semen were diluted to a density of 30 × 10^6^ sperm cells/mL in 100 mL of Beltsville thawing solution^37,61^. During sample transport, semen was maintained at 17 °C in an ice box 61. After arrival in the laboratory, each semen sample was washed with discontinuous 35 and 70% Percoll (Sigma-Aldrich Co, St. Louis, MO, USA) gradient to eliminate non-sperm contents from the semen^62^. Thereafter, sperm pellets were snap-frozen in LN_2_ (− 196 °C) or incubated in RNAlater (12 × 10^7^ sperm cells/mL; Invitrogen, Carlsbad, CA, USA) overnight in 4 °C. Both samples were stored in 4 °C or− 80 °C for 7 days before RNA isolation. To minimize the effect of individual variation, semen samples from three boars were combined and used for method optimization with GAPDH gene. Moreover, to determine whether the optimized method can be used for accurately predicting fertility phenotype of boar spermatozoa, the mRNA expression level of semen from 20 different boars were evaluated.

### Computer-assisted sperm analysis

Sperm motility and motion kinematics of Percoll separated spermatozoa was checked by Computer-assisted sperm analysis system (SAIS-Plus ver.10.1; Medical Supply, Seoul, Korea). Percoll separated spermatozoa was resuspended in 1 mL of mTCM199 media and 10 μL of resuspended sample was placed on pre-heated (37 °C) Makler counting chamber (Sefi Medical Instruments, Haifa, Israel). Under a phase contrast microscope, the motility, hyperactivation, curvilinear velocity, straight line velocity, average path velocity, linearity, beat cross frequency, wobble, and mean amplitude of head lateral displacement of spermatozoa were measured (Supplementary Table S1 online)^32^.

### RNA isolation and cDNA synthesis

Sperm RNAs were isolated by using a PureLink RNA Mini Kit (Invitrogen) according to the manufacturer’s protocol with some modifications. Briefly, spermatozoa pellets were resuspended in PBS and counted under microscope with a Makler counting chamber to adjust the concentration to 40–50 × 10 6 cells/mL. Pellets (40–50 × 10 6 cells) were suspended in non-toxic guanidine-isothiocyanate lysis buffer in PureLink RNA Mini Kit containing β-mercaptoethanol (40 μL/mL; Sigma-Aldrich Co) and homogenized. TRIzol (Invitrogen) and chloroform were added and centrifuged at 12,000 ×  g . The upper layer was transferred into a fresh tube and mixed with pure ethanol (supernatant : pure ethanol = 1:1, v/v; Sigma-Aldrich Co). Sperm RNA was attached to a spin cartridge. For DNase-treated RNA, the spin cartridge membrane was incubated in PureLink DNase mixture (80 µL; Invitrogen) for 15 min at room temperature during washing. Both isolated RNAs with and without DNase were immersed in 20 μL of nuclease-free water. The quality (260/280 ratio) and quantity of sperm RNA were checked with an Epoch Microplate Spectrophotometer (BioTek, Winooski, VT, USA). After matching of total RNA concentration among samples (total 400 ng), cDNA was synthesized with the PrimeScript 1st strand cDNA Synthesis Kit (Takara Bio, Inc., Shiga, Japan) according to the manufacturer’s protocol. Oligo dT and random hexamer primers were compared for cDNA synthesis.

### RT-qPCR

The cDNA was mixed with SYBR Green PCR master mix (Applied Biosystems, Foster city, CA, USA) and amplified on a 7,500 fast real-time PCR system (Applied Biosystems). As a positive control and negative control, cDNA from porcine testis RNA and no template and reverse transcriptase cDNA was used, respectively. All primers were designed based on Reference genome Sscrofa11.1 Primary Assembly. For method optimization, we compared the exon, intron, and exon-exon junction primers of GAPDH, a reference gene for RT-qPCR in boar spermatozoa (Table 2)^63^. The EQTN and PRDX4 genes were selected as target markers of male fertility^37,39^. Exon and intron primers of selected markers were designed; and for reference gene, GAPDH was used (Table 2). The amplification efficiency of primers was tested with calibration curve^64^. Briefly, the cDNA was diluted into 1, 2.5, 5, 10, and 25X (250, 100, 50, 25, and 10 ng) in each well and Cq value of studied genes were analyzed with amount of cDNA (Supplementary Fig. S5 online). The total reaction volume was 20 µL (100 ng of cDNA). The cycling parameters were 95 °C (10 min) followed by 40 cycles at 95 °C (15 s) and 60 °C (60 s). After cycling, continuous melt curve analysis was performed at 95 °C (15 s), 60 °C (60 s), and a progressive increase up to 95 °C (0.5 °C/30 s). Data were analyzed with the 7,500 Software v2.3 (Thermo Fisher Scientific, Waltham, MA, USA). Relative expression of each gene was calculated with delta delta Cq method and to consider the gDNA factor with intron expression, a calculation from Laurell et al. was used^59^.

$${\text{Cq mRNA }} = { } - \log_{2} (2^{ - Cq exon} { }{-}{ }2^{ - Cq intron} )$$

**Table 2:** Designed primers for RT-qPCR.

Primer name	Upper primer (5′–3′)	Tm (°C)	Lower primer (5′–3′)	Tm (°C)	Amplicon size (bp)
GAPDH exon	AAG AGC ACG CGA GGA GGA G	67.1	GGG GTC TGG GAT GGA AAC T	65.1	109
GAPDH Intron	TTC AAG CCC CAG CCA GAT T	66.9	CCG GAA ACA ACC CAA GAC C	66.5	111
GAPDH junction	TCC TGG GCT ACA CTG AGG AC	64.3	CTT GAC GAA GTG GTC GTT GA	64	193
EQTN exon	AAA CCC TGC AAA TGA AGA CAA C	64	CTG CCA AAA TGA TGA CAA AAA G	63.2	106
EQTN intron	GCA GAA CCC CAG TCT CTG TC	63.9	GGG CTC CTT ATC CAA AAT GG	64.2	95
PRDX4 exon	GTG TCC AAC TGA AAT TAT CG	57.1	AGA TGG GTA AAC TGT GAA TC	55.4	101
PRDX4 intron	CGG GCA GAC AAC TCT TAA CAT	63	TGC ACC TTC GAT GAA CTA GC	63	94

All primers were designed genes from pig reference genome Sscrofa11.1 Primary Assembly. Gene accession number: GAPDH (ENSSSCG00000000694); EQTN (ENSSSCG00000005121); PRDX4 (ENSSSCG00000012171).

The size of the PCR products was checked with running gel electrophoresis on 2% agarose gel for 30 min. The gel image was gathered with a PowerShot A640 (Canon, Tokyo, Japan) under ultraviolet light. The image brightness was controlled with PSRemote v1.6.4 software (Breeze Systems, Camberley, UK).

### Fertility data acquisition

Fertility data from randomly selected 20 Yorkshire boars were gathered by AI using their semen. Semen samples were collected after sexual maturation and diluted as described above. AI was performed by professional technicians from Sunjin Co. twice per estrus^32,65,66^. The inseminated sows were managed at 20 ± 5 °C with air circulation, 2:1 light/dark cycle, and adequate feed for pregnant sow during the experimental period. AI was performed for 1 year to avoid the seasonal fluctuation and boar semen was inseminated to average 17.25 ± 1.98 sows (total 360 trial). The average number of born piglets from AI results using boar semen was considered as the fertility outcome.

### Assessment of male fertility predicting power of mRNA

The correlation between fertility and mRNA expression of EQTN and PRDX4 from 20 individual boar semen sample were analyzed with linear regression in all treatments. To estimate the male fertility predicting power of mRNA markers, receiver operating characteristic (ROC) curve analysis was utilized to set the cut-off value and assess the four key parameters *i.e*. sensitivity, specificity, positive predictive value, and negative predictive value of each marker^67^. The cut-off values of relative expression in all treatments were decided based on a fixed litter size (13) to compare each treatment. Sensitivity is the percentage of boars that were true-positive when tested for mRNA expression. Specificity is the percentage of boars that tested as true-negative. The positive predictive value is the percentage of boars that tested as positive and had a true-positive litter size. The negative predictive value is the percentage of boars that tested as negative or had a true-negative litter size.

### Statistics

The Student’s *t*-test, one-way analysis of variance, Pearson correlation test, linear regression test, and ROC curve test were performed with SPSS v1.8 software (SPSS, Inc., Chicago, IL, USA). All data were subjected to a normality test (Shapiro–Wilk test) and homogeneity of variances test (Levene’s test). *P* < 0.05 was considered to indicate significantly different results. All data are expressed as the mean ± SE.

## Supplementary information

Supplementary information (DOCX 1217 kb)
