# Supplementary material for: Optimization of sperm RNA processing for developmental research
Source: Sci Rep. 2020 Jul 14;10:11606. doi: 10.1038/s41598-020-68486-1 (PMC7360572; doi:10.1038/s41598-020-68486-1)
Supplement: Supplementary file 1 — Supplementary information (DOCX 1217 kb) [file 41598_2020_68486_MOESM1_ESM.docx]

**Optimization of sperm RNA processing for developmental research**

Won-Ki Pang, Saehan Kang, Do-Yeal Ryu, Md Saidur Rahman, Yoo-Jin Park, and Myung-Geol Pang^*^

Department of Animal Science & Technology and BET Research Institute, Chung-Ang University, Anseong, Gyeonggi-do, 17546, Republic of Korea

* To whom correspondence should be addressed. Tel: +82.31.670.4841; Fax: +82.31.670.3019; Email: mgpang@cau.ac.kr

**Supplementary Figure**

The figure below is available as supplementary figure,

**Figure S1**: The cDNA synthesis efficiency of oligo dT and random hexamer

**Figure S2**: Oligo dT and random hexamer no template control melting curve analysis in RT-qPCR result from EQTN, PRDX4, and GAPDH.

**Figure S3**: Amplification graph and melting curve analysis of EQTN, PRDX4, and GAPDH RT-qPCR in positive control and spermatozoa.

**Figure S4**: Gel electrophoresis image of RT-qPCR product.

**Figure S5**: The standard curve and qPCR efficiency of studied genes.

**Figure S1**: The cDNA synthesis efficiency of oligo dT and random hexamer. All data are expressed as the mean ± S.E.

**Figure S2**: Oligo dT and random hexamer no template control melting curve analysis in RT-qPCR result from EQTN, PRDX4, and GAPDH.


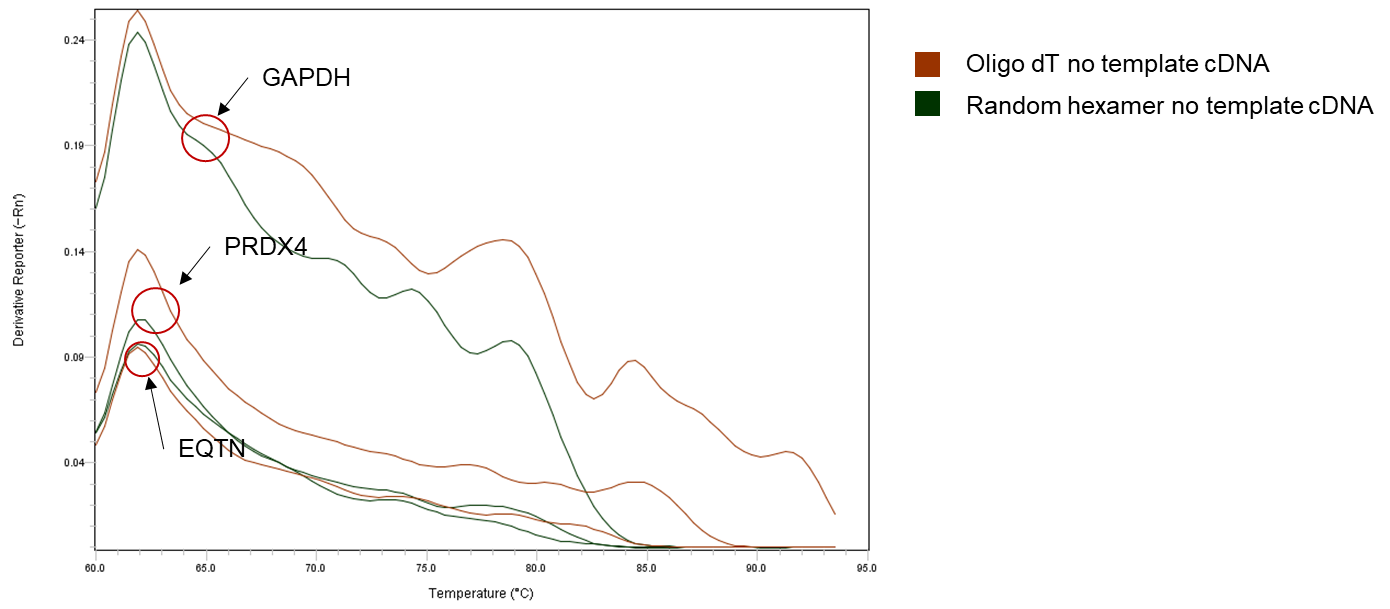


**Figure S3**: Amplification graph and melting curve analysis of EQTN, PRDX4, and GAPDH RT-qPCR in positive control and spermatozoa.


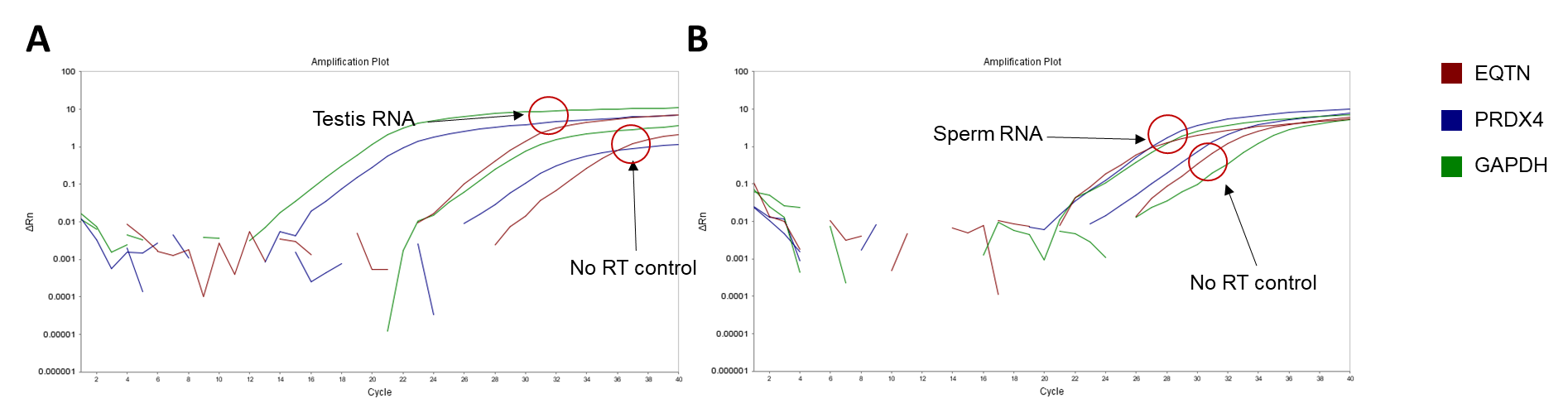


**Figure S4.** Gel electrophoresis image of RT-qPCR product. (A) Oligo dT cDNA, GAPDH RT-qPCR product gel electrophoresis image. (B) Random hexamer cDNA, GAPDH RT-qPCR product gel electrophoresis image.


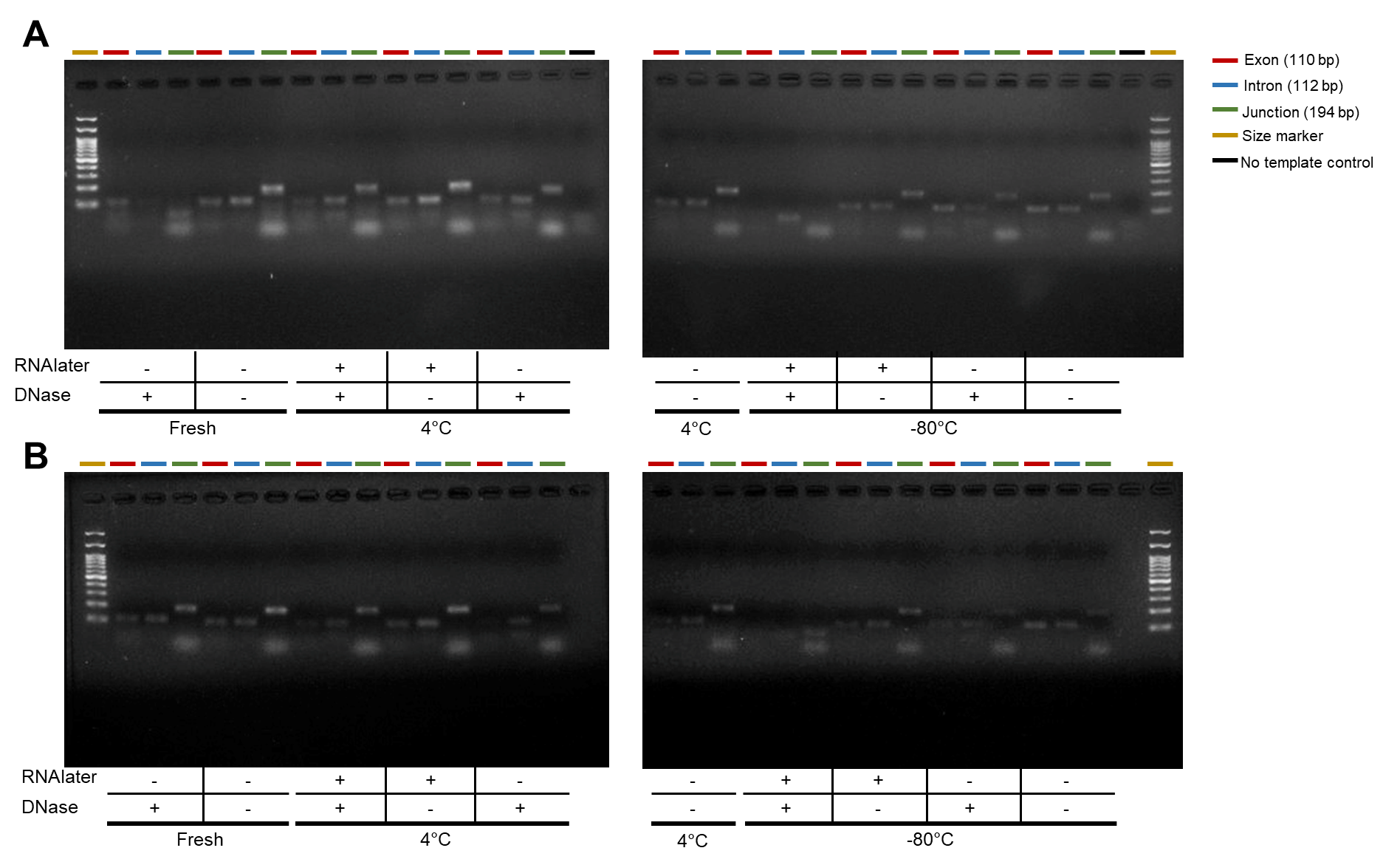


**Figure S5**: The standard curve and qPCR efficiency of studied genes. (A) EQTN standard curve from testis RNA sample. (B) GAPDH standard curve from testis RNA sample. (C) PRDX4 standard curve from testis RNA sample. (D) EQTN standard curve from sperm RNA sample. (E) GAPDH standard curve from sperm RNA sample. (F) PRDX4 standard curve from sperm RNA sample.


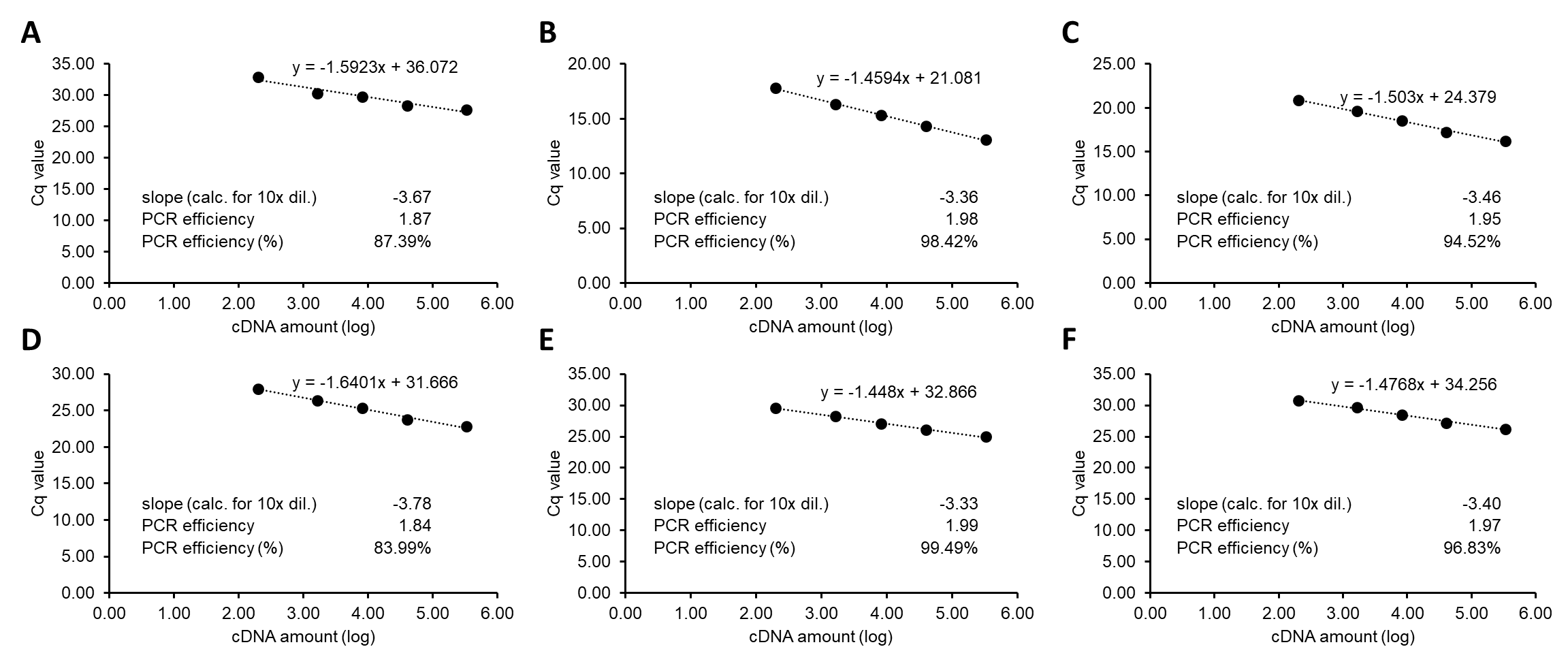


**Supplementary Table**

The table below is available as supplementary table,

**Table S1:** Motility and motion kinematics of spermatozoa after Percoll separation.

**Table S1:** Motility and motion kinematics of spermatozoa after Percoll separation. MOT = motility; HYP = hyperactivation; VCL = curvilinear velocity; VSL = straight line velocity; VAP = average path velocity; LIN = linearity; BCF = beat cross frequency; WOB = wobble; ALH = mean amplitude of head lateral displacement.

| Sample name | MOT (%) | HYP (%) | VCL (μm/s) | VSL (μm/s) | VAP (μm/s) | LIN (%) | BCF (Hz) | WOB (%) | ALH (%) | |
| --- | --- | --- | --- | --- | --- | --- | --- | --- | --- | --- |
| 1 | 89.43 | 25.89 | 164.8 | 75.82 | 85.12 | 45.97 | 10.88 | 51.62 | 7.22 |  |
| 2 | 85.58 | 20.78 | 153.32 | 65.99 | 78.8 | 42.97 | 11.49 | 51.02 | 6.61 |  |
| 3 | 73.63 | 23.08 | 150.28 | 63.76 | 76.59 | 42.43 | 11.9 | 50.98 | 6.57 |  |
| 4 | 94.88 | 36.64 | 181.87 | 75.25 | 91.22 | 41.38 | 10.51 | 50.16 | 7.88 |  |
| 5 | 96.96 | 26.92 | 168.04 | 73.42 | 87.83 | 43.83 | 10.43 | 52.18 | 7.42 |  |
| 6 | 61.76 | 5.07 | 109.76 | 53.12 | 60.61 | 48.23 | 13.02 | 55.13 | 4.89 |  |
| 7 | 71.48 | 7.19 | 136.56 | 80.39 | 82.48 | 58.83 | 12.29 | 60.38 | 6.26 |  |
| 8 | 83.41 | 18.35 | 147.15 | 62.25 | 74.05 | 42.31 | 11.7 | 50.34 | 6.42 |  |
| 9 | 74.25 | 8.65 | 119.74 | 49.1 | 60.32 | 40.98 | 12.95 | 50.37 | 5.3 |  |
| 10 | 72.04 | 12.16 | 141.91 | 72.14 | 81.69 | 50.82 | 11.7 | 57.56 | 6.36 |  |
| 11 | 86.18 | 20.98 | 149.91 | 58.34 | 72.35 | 38.92 | 11.68 | 48.27 | 6.46 |  |
| 12 | 74.23 | 12.28 | 126.78 | 49.74 | 61.25 | 39.23 | 12.61 | 48.3 | 5.54 |  |
| 13 | 74.3 | 11.33 | 130.75 | 52.09 | 63.48 | 39.79 | 12.17 | 48.52 | 5.67 |  |
| 14 | 82.44 | 11.47 | 149.21 | 85.71 | 92.52 | 57.46 | 10.96 | 62 | 7.02 |  |
| 15 | 80.21 | 13.11 | 133.35 | 55.34 | 67.64 | 41.43 | 11.94 | 50.7 | 5.83 |  |
| 16 | 79.01 | 13.24 | 131.61 | 48.38 | 62.19 | 36.76 | 12.44 | 47.25 | 5.79 |  |
| 17 | 89.72 | 12.91 | 151.21 | 77.06 | 86.01 | 50.95 | 11.91 | 56.89 | 6.9 |  |
| 18 | 86.58 | 14.84 | 142.33 | 60.69 | 75.6 | 42.63 | 12.17 | 53.12 | 6.19 |  |
| 19 | 85.95 | 17.26 | 160.04 | 87.17 | 93.66 | 51.46 | 10.84 | 58.5 | 7.37 |  |
| 20 | 64.71 | 9.78 | 118.02 | 48.84 | 58.74 | 41.43 | 12.98 | 49.79 | 5.19 |  |
